# Herpes simplex virus: global infection prevalence and incidence estimates, 2016

**DOI:** 10.2471/BLT.19.237149

**Published:** 2020-03-25

**Authors:** Charlotte James, Manale Harfouche, Nicky J Welton, Katherine ME Turner, Laith J Abu-Raddad, Sami L Gottlieb, Katharine J Looker

**Affiliations:** a Population Health Sciences, Bristol Medical School, University of Bristol, Bristol, Oakfield House, Oakfield Grove, Bristol BS8 2BN, England.; b Weill Cornell Medical College, Doha, Qatar.; c Bristol Veterinary School, University of Bristol, Bristol, England.; d Department of Reproductive Health and Research, World Health Organization, Geneva, Switzerland.

## Abstract

**Objective:**

To generate global and regional estimates for the prevalence and incidence of herpes simplex virus (HSV) type 1 and type 2 infection for 2016.

**Methods:**

To obtain data, we undertook a systematic review to identify studies up to August 2018. Adjustments were made to account for HSV test sensitivity and specificity. For each World Health Organization (WHO) region, we applied a constant incidence model to pooled prevalence by age and sex to estimate the prevalence and incidence of HSV types 1 and 2 infections. For HSV type 1, we apportioned infection by anatomical site using pooled estimates of the proportions that were oral and genital.

**Findings:**

In 2016, an estimated 491.5 million people (95% uncertainty interval, UI: 430.4 million–610.6 million) were living with HSV type 2 infection, equivalent to 13.2% of the world’s population aged 15–49 years. An estimated 3752.0 million people (95% UI: 3555.5 million–3854.6 million) had HSV type 1 infection at any site, equivalent to a global prevalence of 66.6% in 0–49-year-olds. Differing patterns were observed by age, sex and geographical region, with HSV type 2 prevalence being highest among women and in the WHO African Region.

**Conclusion:**

An estimated half a billion people had genital infection with HSV type 2 or type 1, and several billion had oral HSV type 1 infection. Millions of people may also be at higher risk of acquiring human immunodeficiency virus (HIV), particularly women in the WHO African Region who have the highest HSV type 2 prevalence and exposure to HIV.

## Résumé

**Objectif:**

Estimer la prévalence et l'incidence, au niveau régional et mondial, des infections au virus Herpes simplex (HSV) de type 1 et de type 2 en 2016.

**Méthodes:**

Pour nous procurer les données nécessaires, nous avons entrepris une revue systématique afin d'identifier des études publiées en août 2018 au plus tard. Des ajustements ont été effectués pour tenir compte de la sensibilité et de la spécificité du dépistage HSV. Pour chaque région définie par l'Organisation mondiale de la Santé (OMS), nous avons appliqué un modèle d'incidence constante par groupe de prévalence en fonction de l'âge et du sexe. Notre but était d'estimer la prévalence et l'incidence des infections à HSV de type 1 et 2. Pour le HSV de type 1, nous avons réparti les infections selon le site anatomique, en utilisant des estimations groupées de la proportion d'atteintes orales et génitales.

**Résultats:**

En 2016, environ 491,5 millions de personnes (intervalle d'incertitude de 95%: 430,4 millions–610,6 millions) vivaient avec une infection à HSV de type 2, l'équivalent de 13,2 % de la population mondiale entre 15 et 49 ans. Nous estimons que 3752,0 millions de personnes (intervalle d'incertitude de 95%: 3555,5 millions–3854,6 millions) souffraient d'une infection à HSV de type 1 à un site quelconque, ce qui représente une prévalence globale de 66,6% chez les 0–49 ans. Différentes tendances ont été constatées en fonction de l'âge, du sexe et de la zone géographique, le plus fort taux de prévalence de HSV de type 2 étant observé chez les femmes de la région Afrique de l'OMS.

**Conclusion:**

Près d'un demi-milliard de personnes présentaient une infection génitale à HSV de type 2 ou type 1, et plusieurs milliards avaient une infection orale à HSV de type 1. Des millions de personnes pourraient également être plus susceptibles de contracter le virus de l'immunodéficience humaine (VIH), en particulier les femmes de la région Afrique de l'OMS qui possèdent la plus grande prévalence de HSV de type 2 et sont davantage exposées au VIH.

## Resumen

**Objetivo:**

Calcular las estimaciones mundiales y regionales de la prevalencia y la incidencia de la infección por el virus del herpes simple (VHS) tipo 1 y tipo 2 para 2016.

**Métodos:**

Se realizó una revisión sistemática para identificar los estudios hasta agosto de 2018 con el fin de obtener datos. Se hicieron ajustes para tener en cuenta la sensibilidad y la especificidad de la prueba de VHS. Se aplicó un modelo de incidencia constante para cada región de la Organización Mundial de la Salud (OMS) con el fin de agrupar la prevalencia por edad y sexo para estimar la prevalencia y la incidencia de las infecciones por VHS de los tipos 1 y 2. Para el VHS tipo 1, se repartió la infección por sitio anatómico utilizando estimaciones agrupadas de los porcentajes que eran orales y genitales.

**Resultados:**

En 2016, se estima que 491,5 millones de personas (95 % de intervalo de incertidumbre, UI: 430,4 millones a 610,6 millones) vivían con la infección por el VHS tipo 2, equivalente al 13,2 % de la población mundial de 15 a 49 años. Se estima que 3752,0 millones de personas (95 % UI: 3555,5 millones a 3854,6 millones) tenían la infección por VHS tipo 1 sin importar el lugar, lo que equivale a una prevalencia mundial del 66,6 % en las personas de 0 a 49 años. Se observaron patrones diferentes según la edad, el sexo y la región geográfica, siendo la mayor prevalencia del VHS tipo 2 entre las mujeres y en la región de África de la OMS.

**Conclusión:**

Se estima que 500 millones de personas tienen una infección genital con VHS tipo 2 o tipo 1, y varios miles de millones tienen una infección oral de VHS tipo 1. Millones de personas también pueden correr un mayor riesgo de contraer el virus de la inmunodeficiencia humana (VIH), en particular las mujeres de la Región de África de la OMS que tienen la mayor prevalencia del VHS tipo 2 y la mayor exposición al VIH.

## ملخص

الغرض وضع تقديرات للانتشار والإصابة العالمية والإقليمية بعدوى فيروس الهربس البسيط (HSV)، النوعين الأول والثاني في عام 2016.

الطريقة للحصول على بيانات، قمنا بإجراء مراجعة لتحديد الدراسات حتى أغسطس/آب 2018. كما تم إجراء تعديلات لتناسب حساسية ومدى خصوصية اختبار فيروس HSV. لكل منطقة من مناطق منظمة الصحة العالمية (WHO)، قمنا بتطبيق نموذج حدوث ثابت على حالات الانتشار المصنفة حسب العمر والجنس، وذلك لتقدير انتشار وحدوث العدوى بفيروس HSV من النوعين الأول والثاني. بالنسبة للنوع الأول من فيروس HSV، قمنا بتقسيم العدوى إلى نسب حسب الموقع التشريحي، باستخدام تقديرات مجمعة للنسب التي كانت عن طريق الفم أو الأعضاء التناسلية.

النتائج في علم 2016، كان ما يقدر بـ 491.5 مليون شخص (فاصل عدم الثقة 95%: 430.4 مليون إلى 610.6 مليون) يعيشون وهم مصابون بعدوي فيروس HSV من النوع الثاني، أي ما يعادل 13.2% من سكان العالم الذين تتراوح أعمارهم بين 15 و49 عاماً. وما يقدر بـ 3752 مليون شخص (فاصل عدم ثقة 95%: 3555.5 مليون إلى 610.6 مليون) كان لديهم عدوي فيروس HSV من النوع الأول في أي موقع، أي ما يعادل انتشار عالمي بنسبة 66.6% من الأشخاص الذين تتراوح أعمارهم من سن الولادة إلى 49 عاماً. وتمت ملاحظة أنماط متباينة حسب العمر والجنس والمنطقة الجغرافية، مع وجود أعلى انتشار لفيروس HSV من النوع الثاني بين النساء، وفي المنطقة الأفريقية التابعة لمنظمة الصحة العالمية (WHO).

الاستنتاج تم تقدير أن قرابة نصف مليار شخص كانوا مصابين بعدوى فيروس HSV من النوع الثاني أو الأول في المنطقة التناسلية، مع إصابة عدة مليارات من الأشخاص بعدوى فيروس HSV من النوع الأول عن طريق الفم. قد يكون الملايين من الأشخاص أيضاً أكثر عرضة لخطر الإصابة بفيروس نقص المناعة البشرية (HIV)، وخاصة النساء في المنطقة الأفريقية التابعة لمنظمة الصحة العالمية (WHO)، والذين لديهم أعلى معدل لانتشار فيروس HSV من النوع الثاني، والتعرض للإصابة بفيروس HIV.

## 摘要

**目标:**

旨在估计 2016 年全球和区域范围内感染单纯疱疹病毒 (HSV) 1 型和 2 型的患病率和发病率。

**方法:**

为了收集数据，我们选取截至 2018 年 8 月的研究进行系统性回顾。对单纯疱疹病毒 (HSV) 检测的敏感性和特异性进行了适当调整。对于世界卫生组织 (WHO) 的每个地区，我们利用恒定发病率模型，按年龄和性别混合患病率，以估计感染单纯疱疹病毒 1 型和 2 型的患病率和发病率。对于单纯疱疹病毒 1 型，我们使用口腔和生殖器比例的混合估计数，按解剖部位来分配感染。

**结果:**

2016 年，估计有 4.915 亿人（95% 不确定区间，UI：4.304 亿-6.106 亿）人感染单纯疱疹病毒 2 型，占世界 15-49 岁人口的 13.2%。估计有 37.52 亿人（95% UI：35.555 亿-38.546 亿）在世界各地感染单纯疱疹病毒 1 型，相当于全球 66.6% 的 0-49 岁人群中的患病率。按年龄、性别和地理区域观察到不同的模式，其中单纯疱疹病毒 2 型患病率在妇女和世卫组织非洲地区最高。

**结论:**

据估计，5 亿人患有单纯疱疹病毒 2 型或 1 型生殖器感染，另外有几十亿人患有单纯疱疹病毒 1 型口腔感染。数百万人还可能有更高风险感染人类免疫缺陷病毒 (HIV)，尤其是世卫组织非洲地区的妇女，她们的单纯疱疹病毒 2 型患病率和接触人类免疫缺陷病毒 (HIV) 的可能性最高。

## Резюме

**Цель:**

Оценка глобальных и региональных показателей распространенности и заболеваемости вирусом простого герпеса (HSV) 1-го и 2-го типов по состоянию на 2016 год.

**Методы:**

Для получения данных был проведен систематический обзор и выявлены соответствующие исследования вплоть до августа 2018 года. Данные были скорректированы с учетом чувствительности и специфики тестов на HSV. Для каждого из регионов по классификации Всемирной организации здравоохранения (ВОЗ) авторы применили модель постоянной заболеваемости в отношении общей распространенности по полу и возрасту для оценки распространенности и заболеваемости HSV 1-го и 2-го типов. Для HSV 1-го типа авторы разделили инфицированных по анатомическому участку с использованием обобщенных оценок пропорций орального и генитального герпеса.

**Результаты:**

По состоянию на 2016 год оценочное количество инфицированных HSV 2-го типа составило 491,5 млн (95%-й интервал неопределенности, ИН: 430,4–610,6 млн), что составляет 13,2% от мировой популяции лиц в возрасте 15–49 лет. Примерно 3752,0 миллиона человек (95%-й ИН: 3555,5–3854,6 млн) были инфицированы HSV 1-го типа в каком-либо из участков организма, что эквивалентно коэффициенту глобальной распространенности, равному 66,6% среди населения в возрасте от 0 до 49 лет. Наблюдались различия в распределении заболеваемости по возрастам, полу и географическим регионам, при этом HSV 2-го типа преобладал среди женщин и в Африканском регионе ВОЗ.

**Выводы:**

Примерно полмиллиарда людей имеет генитальную инфекцию HSV 2-го или 1-го типа, а оральную инфекцию HSV 1-го типа имеет несколько миллиардов человек. Миллионы людей также могут быть подвержены более высокому риску заражения вирусом иммунодефицита человека (ВИЧ), в частности женщины из Африканского региона ВОЗ, среди которых наблюдается самый высокий уровень распространенности HSV 2-го типа и которые сильнее всего подвергаются риску ВИЧ-инфекции.

## Introduction

Herpes simplex virus (HSV) infections are widespread among humans globally.^1^^,^^2^ The infection is lifelong and characterized by periodic reactivations at the infection site. HSV type 1 is primarily transmitted by oral-to-oral contact and commonly causes orolabial herpes (cold sores).^3^ Type 1 virus also causes rarer conditions, such as keratitis and other ocular sequalae, and encephalitis.^4^ HSV type 1 genital infection from oral-to-genital contact is becoming increasingly common, although reactivations are less frequent than for HSV type 2.^5^^–^^10^ HSV type 2 is almost entirely sexually transmitted, causing genital herpes.^11^ Genital HSV infection may be unrecognized or result in painful genital ulcer disease in a proportion of those infected. Neonates can acquire HSV infection from genitally infected mothers during birth and from oral contact with caregivers postnatally.^12^ Neonatal infection, although rare, has a high fatality and disability rate in surviving infants.^12^ Evidence also suggests that HSV type 2 infection increases the risk of acquiring human immunodeficiency virus (HIV).^13^ Symptomatic and asymptomatic viral shedding are common for both HSV type 1 and type 2.^14^^,^^15^ Thus, infected individuals can be asymptomatic yet infectious, allowing these viruses to be transmitted unknowingly, a factor which contributes to the large global prevalence of HSV infection.

The World Health Organization (WHO) has produced global and regional estimates of HSV type 2 infection prevalence and incidence (derived from prevalence) among individuals 15–49 years or age twice before: for the years 2005 and 2012.^2^^,^^16^ The first estimates of HSV type 1 infection at any site in those aged 0–49 years of age, and of genital HSV type 1 infection in those aged 15–49 years of age, were done for 2012.^1^ The Global Burden of Disease (GBD) study has also produced estimates for HSV type 2 infection (again deriving incidence from estimated prevalence, similar to the WHO estimates), most recently for 2017.^17^ However, these estimates are not directly comparable to the WHO estimates as they extend to age 99 years of age, are not adjusted for assay performance and use different regional groupings than the WHO estimates. Furthermore, the GBD study does not produce any estimates for HSV type 1 infection, an increasingly important cause of genital infection.

Estimates of HSV infection across geographical regions, age, sex, HSV type and infection site (oral versus genital) are needed for advocacy and resource planning. In 2016, the World Health Assembly adopted the Global Health Sector Strategy on Sexually Transmitted Infections, 2016–2021,^18^ which aims to end sexually transmitted infections as a public health concern by 2030. The strategy sets out reduction targets, which in turn depend on reliable baseline estimates for each sexually transmitted infection. Quantifying HSV infection and disease is also necessary to guide the development of new products, such as vaccines.^19^^–^^21^ Infection estimates can be used as a starting point for estimating the burden of HSV-related disease when direct incidence data are lacking, by applying the risks of particular outcomes to the number of people infected, as has been done for neonatal herpes.^22^ In this systematic review we made global and regional estimates of HSV type 2 and genital HSV type 1 infection for the year 2016, incorporating newly available data, and estimates specifically for oral HSV type 1 infection.

## Methods

We used similar methods as for our previous estimates.^1^^,^^2^^,^^16^ We conducted a systematic literature search followed by pooling of extracted data using meta-analysis. First, we searched the online databases MEDLINE® and Embase® to identify relevant studies with publication dates between August 2013 (to ensure overlap with the literature searches informing the 2012 estimates) and August 2018. We included studies of the prevalence and incidence of HSV type 1 and type 2 infection, as measured by the detection of type-specific immunoglobulin G antibodies, in any language. We applied broad inclusion and exclusion criteria to the studies to extract the data and then applied additional criteria to the extracted data for calculating the estimates. We excluded high-risk populations and based the calculations on prevalence data from general populations only. Incidence data were used solely for comparison and validation purposes. Further details of the search strategy, data extraction and synthesis, and definitions of general populations are in the data repository.^23^

We then pooled the newly extracted data with data from our previous estimates, using studies from year 2004 or later. Thus, there was a large overlap in the studies included between the current and previous set of estimates. We pooled prevalence values by sex where possible and 5-year age groups for each WHO region (African, Americas, Eastern Mediterranean, Europe, South-East Asia and Western Pacific) and separately for HSV type 1 and type 2. The force of the infection was then calibrated to each set of pooled prevalence values over age, assuming a constant force of infection with age. Before pooling, we adjusted the prevalence values for the sensitivity and specificity of the tests used to detect HSV in different studies (data repository).^23^ Smoothed prevalence and derived incidence (that is, from the calibrated force of infection) were applied to population data for 2016^24^ to obtain the most up-to-date estimates of the number of people with prevalent (existing) and incident (newly-acquired over one year) HSV type 1 and 2 infection by WHO region. Estimates for oral HSV type 1 infection were done for individuals 0–49 years of age, and estimates for HSV type 2 and genital HSV type 1 infection were done for individuals 15–49 years of age. We also did a speculative analysis to estimate the number of older individuals infected by applying the prevalence in those 45–49 years of age to population numbers for those 50–99 years of age. Further details of the calculation of prevalence, incidence and uncertainty bounds are in the data repository.^23^

To estimate the proportion of individuals infected at different sites, we first pooled values from longitudinal studies of the proportions of adults with oral (pooled estimate: 36.4%) and genital (pooled estimate: 72.4%) symptoms among all adults with HSV type 1 seroconversion, which was accompanied by symptoms (either or both sites).^25^^–^^28^ In other words, we estimated the proportions of new HSV type 1 infections that were oral versus genital for individuals where the site of infection could be determined on the basis of symptoms. Pooling was done using the *metan* command in Stata, version 16 (StataCorp, College Station, United States of America) and assuming a random effects model. We then applied these proportions to HSV type 1 incidence only in those 15–49 years of age to estimate the numbers with oral and genital HSV type 1 infection separately. This method was slightly different to the method of estimating genital HSV type 1 infection for 2012, in which values from two studies of symptomatic HSV type 1 seroconversions were used to generate two separate sets of estimates in adults.^1^ HSV type 1 infection in those younger than 15 years was assumed to be all oral. In a separate sensitivity analysis, we limited those able to be infected with genital HSV type 1 to the proportion of individuals by age who engaged in oral sex in the last 12 months (data for females and males combined) according to the National Health and Nutrition Evaluation Survey 2015–2016, the largest, national population-based survey in the USA.^29^ We calculated the total percentage of people with genital infection due to either HSV type 1 by summing the prevalence of each infection, and then adjusting for the percentage of people assumed to be genitally infected with both viral types.

## Results

### Literature search

**Fig. 1:**
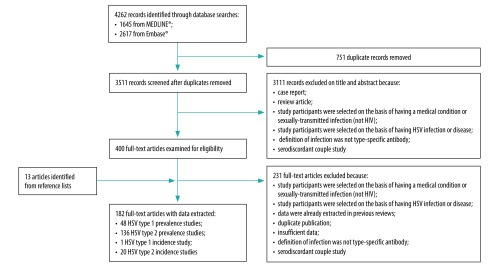
Flowchart on the selection of studies for estimating infection prevalence and incidence of herpes simplex virus, 2016

HIV: human immunodeficiency virus; HSV: herpes simplex virus.

We identified a total of 4262 publications in the updated literature search (Fig. 1). After removal of duplicates, we screened 3511 records on the basis of title and abstract, and excluded a further 3111 records, which did not meet the criteria for relevance. We obtained full texts for the remaining 400 records along with an additional 13 publications identified from reference lists.^8^^,^^30^^,^^31^ Of these 413 publications, 182 contained relevant data and were subsequently included in the data extraction: 48 HSV type 1 prevalence studies, 136 HSV type 2 prevalence studies, 1 HSV type 1 incidence study and 20 HSV type 2 incidence studies (some studies contributed data in more than one category). However, not all of these studies met our criteria for inclusion in the estimates, while some studies identified in previous reviews were still sufficiently recent (after 2004). 

Pooling data from all the studies meeting our criteria we made the estimates using 474 HSV type 2 prevalence data points (262 from newly identified studies) and 223 HSV type 1 prevalence data points (128 from newly identified studies). The number of data points by age and sex, the countries contributing data and the inclusion criteria applied is available in the data repository.^23^

In comparison with the 2012 estimates, the number of available prevalence data points for 2016 improved for both HSV types 1 and 2. However, this increase did not generally follow from an increase in the number of countries represented, as the number of contributing countries mostly declined between the 2012 and 2016 estimates. The decline was particularly apparent for the WHO Region of the Americas, where HSV type 1 estimates for males were based solely on data from individuals in the USA. The prevalence and incidence data from studies newly extracted for this review^32^^–^^122^ are shown in the data repository.^23^

### Prevalence of infection

**Fig. 2:**
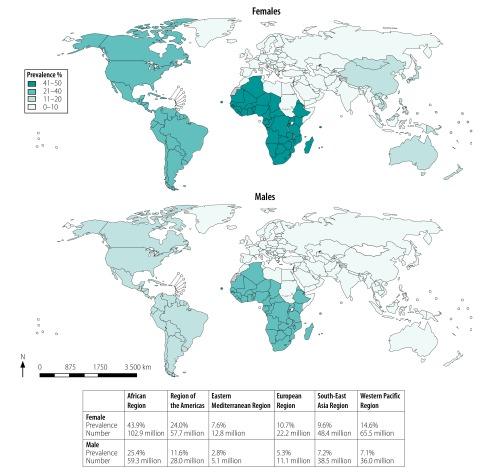
Map of regional estimates of the number and prevalence of herpes simplex virus type 2 infections in females and males, 2016

Notes: Numbers are the year 2016 estimates of the number of people living aged 15–49 years of age with herpes simplex virus type 2 infection. Prevalences are the percentage of infected people in the age- sex- and region-specific population. Regions are World Health Organization definitions. Global estimates are presented in Table 1.

Our estimates for 2016 found that a total of 491.5 million (95% UI: 430.4 million–610.6 million) individuals 15–49 years of age worldwide were living with HSV type 2 infection (Table 1 and Fig. 2). More women (313.5 million) than men (178.0 million) were infected. The number of people infected was highest in the WHO African Region (102.9 million females and 59.3 million males), followed by the Western Pacific, South-East Asia and Americas Regions. The estimated prevalence of HSV type 2 in the global population of 3735.6 million people 15–49 years of age was 13.2% (95% UI: 11.5–16.3) and was highest among the population of the African Region, followed by the Region of the Americas, and among women. The number infected increased with age, largely mirroring increases in the prevalence with age, although differences in population sizes also affected the observed numbers. The regional pooled prevalence values and model fits for HSV type 2 infection is available in the data repository.^23^

**Table 1:** Global and regional estimates of the prevalence of herpes simplex virus type 2 infections by age and sex, 2016

WHO region by sex	No. of infected people in millions (population prevalence, %) by age group								95% UI^a^
	15–19 years	20–24 years	25–29 years	30–34 years	35–39 years	40–44 years	45–49 years	Total	
**Total**	27.8 (4.8)	49.6 (8.5)	68.6 (11.4)	78.9 (14.3)	83.3 (16.8)	89.6 (18.8)	93.7 (20.8)	491.5 (13.2)	430.4–610.6 (11.5–16.3)
**Female**									
Africa	10.1 (21.7)	15.3 (35.9)	17.5 (46.5)	17.8 (54.2)	16.3 (60.0)	14.1 (64.2)	11.9 (67.3)	102.9 (43.9)	85.0–120.0 (36.3–51.2)
Americas	2.6 (7.8)	5.1 (14.4)	7.3 (20.5)	9.1 (26.1)	10.3 (31.4)	11.3 (36.3)	12.0 (40.8)	57.7 (24.0)	46.4–71.6 (19.3–29.7)
Eastern Mediterranean	0.7 (2.5)	1.3 (4.6)	1.9 (6.8)	2.3 (8.8)	2.3 (10.9)	2.2 (12.9)	2.2 (14.8)	12.8 (7.6)	5.7–29.4 (3.4–17.6)
Europe	0.7 (3.0)	1.5 (5.7)	2.5 (8.3)	3.4 (10.8)	4.0 (13.2)	4.8 (15.6)	5.4 (17.9)	22.2 (10.7)	10.4–45.2 (5.0–21.7)
South-East Asia	2.3 (3.0)	4.4 (5.6)	6.3 (8.2)	7.8 (10.7)	8.7 (13.1)	9.3 (15.5)	9.5 (17.8)	48.4 (9.6)	20.2–105.2 (4.0–20.9)
Western Pacific	2.0 (4.2)	4.7 (7.8)	8.7 (11.3)	10.0 (14.7)	10.9 (17.9)	14.9 (21.0)	18.3 (24.0)	69.5 (14.6)	43.8–106.3 (9.2–22.3)
Total	18.4 (6.6)	32.2 (11.4)	44.1 (15.0)	50.3 (18.5)	52.7 (21.6)	56.5 (24.1)	59.2 (26.4)	313.5 (17.1)	265.7–389.1 (14.5–21.3)
**Male**									
Africa	4.6 (9.8)	7.6 (17.8)	9.4 (25.1)	10.3 (31.8)	10.1 (37.9)	9.3 (43.4)	8.1 (48.5)	59.3 (25.4)	44.1–77.1 (18.9–33.0)
Americas	1.2 (3.6)	2.5 (6.7)	3.5 (9.7)	4.4 (12.6)	5.0 (15.5)	5.5 (18.2)	5.9 (20.8)	28.0 (11.6)	18.2–42.0 (7.5–17.3)
Eastern Mediterranean	0.2 (0.9)	0.5 (1.6)	0.7 (2.4)	0.9 (3.2)	0.9 (4.0)	0.9 (4.7)	0.9 (5.5)	5.1 (2.8)	1.1–23.9 (0.6–13.2)
Europe	0.3 (1.5)	0.8 (2.8)	1.3 (4.1)	1.7 (5.4)	2.0 (6.6)	2.4 (7.9)	2.7 (9.1)	11.1 (5.3)	5.1–23.1 (2.4–11.0)
South-East Asia	1.9 (2.2)	3.6 (4.2)	5.0 (6.2)	6.2 (8.1)	6.9 (10.0)	7.3 (11.8)	7.5 (13.7)	38.5 (7.2)	12.2–117.6 (2.3–22.1)
Western Pacific	1.1 (2.0)	2.5 (3.7)	4.5 (5.5)	5.1 (7.2)	5.6 (8.8)	7.8 (10.5)	9.4 (12.1)	36.0 (7.1)	15.8–79.7 (3.1–15.7)
Total	9.4 (3.1)	17.3 (5.8)	24.4 (7.9)	28.6 (10.2)	30.6 (12.2)	33.1 (13.8)	34.5 (15.2)	178.0 (9.3)	140.6–270.1 (7.4–14.2)

UI: uncertainty interval; WHO: World Health Organization.

^a^ 95% UI of the total no. of infected people in millions (95% UI of percentage prevalence).

Notes: Numbers are the year 2016 estimated number of people living with herpes simplex virus type 2 infection. Prevalences are the percentage of infected people in the age- sex- and region-specific population. Numbers do not always sum exactly to the totals due to rounding. Regions are World Health Organization definitions.

An estimated 3583.5 million (95% UI: 3322.2 million–3715.8 million) of the 5632.6 million global population 0–49 years of age were infected orally with HSV type 1, a prevalence of 63.6% (95% UI: 59.0–66.0; Table 2). The number of people with oral HSV-1 was largest in the WHO South-East Asia Region, followed by the Western Pacific Region. Genital HSV type 1 infection affected an estimated 192.0 million (95% UI: 123.0 million–294.0 million) individuals 15–49 years of age worldwide (Table 3), equivalent to a prevalence of 5.2% (95% UI: 3.3–8.0). The number of people with genital HSV type 1 was highest in the Region of the Americas, followed by the European Region. There was a general trend of increasing numbers of people infected with both oral and genital HSV type 1 infection by age, mirroring the increasing prevalence of both infections in our model. 

**Table 2:** Global and regional estimates of the prevalence of oral herpes simplex virus type 1 infection by age and sex, 2016

WHO region by sex	No. of infected people in millions (population prevalence, %) by age group											95% UI^a^
	0–4 years	5–9 years	10–14 years	15–19 years	20–24 years	25–29 years	30–34 years	35–39 years	40–44 years	45–49 years	Total	
**Total**	181.0 (27.4)	371.4 (58.5)	404.3 (67.1)	404.0 (69.7)	407.7 (70.0)	423.3 (70.5)	389.2 (70.5)	348.0 (70.4)	335.9 (70.6)	318.6 (70.7)	3583.5 (63.6)	3322.2–3715.8 (59.0–66.0)
**Female**												
Africa	37.9 (64.9)	62.9 (93.7)	57.4 (96.9)	49.8 (97.2)	43.0 (97.3)	37.4 (97.3)	32.3 (97.3)	26.7 (97.3)	21.5 (97.3)	17.3 (97.3)	386.2 (87.8)	354.8–400.9 (80.6–91.1)
Americas	2.1 (7.6)	6.9 (21.1)	11.2 (32.5)	14.3 (39.3)	15.7 (42.3)	16.4 (44.9)	16.7 (47.1)	16.3 (48.9)	15.8 (50.5)	15.4 (51.9)	130.8 (37.8)	121.8–140.6 (35.2–40.6)
Eastern Mediterranean	6.4 (20.9)	16.7 (49.6)	20.6 (66.8)	21.8 (74.3)	21.9 (76.6)	22.2 (78.0)	20.6 (78.8)	17.4 (79.3)	14.0 (79.6)	11.8 (79.7)	173.5 (63.3)	112.0–220.9 (40.9–80.7)
Europe	3.6 (17.2)	10.4 (42.1)	13.9 (58.1)	15.9 (65.6)	18.5 (68.0)	21.8 (69.6)	22.5 (70.6)	22.2 (71.3)	22.2 (71.7)	22.0 (72.0)	173.1 (60.6)	126.6–210.8 (44.4–73.9)
South-East Asia	23.5 (37.5)	51.4 (61.9)	57.5 (66.7)	57.3 (67.5)	55.2 (67.6)	53.3 (67.6)	50.7 (67.6)	45.9 (67.6)	41.0 (67.6)	36.6 (67.6)	472.5 (62.3)	449.4–485.5 (59.3–64.0)
Western Pacific	15.3 (35.0)	35.6 (67.2)	40.9 (78.1)	43.4 (80.9)	51.3 (81.4)	64.6 (81.5)	56.8 (81.6)	50.7 (81.6)	58.9 (81.6)	62.9 (81.6)	480.4 (74.8)	332.7–517.9 (51.8–80.6)
Total	88.9 (27.9)	184.0 (60.0)	201.5 (69.3)	202.4 (72.2)	205.6 (72.8)	215.8 (73.6)	199.7 (73.5)	179.1 (73.4)	173.4 (73.8)	165.9 (74.1)	1816.5 (66.1)	1641.5–1899.6 (59.8–69.2)
**Male**												
Africa	39.0 (64.9)	64.4 (93.7)	58.5 (96.9)	50.5 (97.2)	43.3 (97.3)	37.3 (97.3)	32.0 (97.3)	26.4 (97.3)	21.1 (97.3)	16.5 (97.3)	389.0 (87.6)	356.9–404.1 (80.4–91.0)
Americas	1.7 (6.1)	5.9 (17.2)	9.7 (27.0)	12.4 (33.0)	13.7 (35.7)	14.3 (38.2)	14.3 (40.3)	13.9 (42.2)	13.4 (43.9)	13.0 (45.4)	112.3 (31.9)	103.8–120.4 (29.5–34.2)
Eastern Mediterranean	6.8 (20.9)	17.6 (49.6)	21.8 (66.8)	23.2 (74.3)	23.3 (76.6)	23.8 (78.0)	22.1 (78.8)	19.0 (79.3)	15.8 (79.6)	13.2 (79.7)	186.6 (63.5)	120.6–237.3 (41.1–80.8)
Europe	1.8 (7.9)	5.7 (22.0)	8.4 (33.9)	10.4 (40.9)	12.5 (44.0)	15.0 (46.6)	15.8 (48.9)	15.7 (50.8)	16.0 (52.4)	15.9 (53.7)	117.1 (40.1)	102.2–134.7 (35.0–46.1)
South-East Asia	25.6 (37.5)	56.2 (61.9)	63.1 (66.7)	62.6 (67.5)	59.7 (67.6)	56.5 (67.6)	53.0 (67.6)	47.5 (67.6)	42.5 (67.6)	37.7 (67.6)	504.3 (62.2)	479.3–518.4 (59.1–63.9)
Western Pacific	17.1 (34.8)	37.7 (62.2)	41.3 (69.6)	42.6 (71.2)	49.5 (71.4)	60.5 (71.5)	52.3 (71.5)	46.4 (71.5)	53.8 (71.5)	56.5 (71.5)	457.6 (66.0)	301.0–488.0 (43.4–70.4)
Total	92.1 (27.0)	187.4 (57.1)	202.8 (65.0)	201.6 (67.3)	202.1 (67.4)	207.4 (67.5)	189.5 (67.5)	168.9 (67.4)	162.5 (67.5)	152.7 (67.4)	1767.0 (61.2)	1594.7–1837.6 (55.2–63.7)

UI: uncertainty interval; WHO: World Health Organization.

^a^ 95% UI of the total no. of infected people in millions (95% UI of percentage prevalence).

Notes: Numbers are the year 2016 estimated number of people living with oral herpes simplex virus type 1 infection. Prevalences are the percentage of infected people in the age- sex- and region-specific population. Numbers do not always sum exactly to the totals due to rounding. Regions are World Health Organization definitions.

**Table 3:** Global and regional estimates of the prevalence of genital herpes simplex virus type 1 infection by age and sex, 2016

WHO region by sex	No. of infected people in millions (population prevalence, %) by age								95% UI^a^
	15–19 years	20–24 years	25–29 years	30–34 years	35–39 years	40–44 years	45–49 years	Total	
**Total**	5.8 (1.0)	17.9 (3.1)	27.5 (4.6)	32.6 (5.9)	34.7 (7.0)	36.3 (7.6)	37.2 (8.2)	192.0 (5.2)	123.0–294.0 (3.3–8.0)
**Female**									
Africa	< 0.1^b^ (0.1)	< 0.1^b^ (0.1)	< 0.1^b^ (0.1)	< 0.1^b^ (0.1)	< 0.1^b^ (0.1)	< 0.1^b^ (0.1)	< 0.1^b^ (0.1)	0.2 (0.1)	< 0.1^c^–3.8 (0.0–1.8)
Americas	1.0 (3.4)	3.3 (9.4)	5.2 (14.5)	6.6 (18.9)	7.4 (22.6)	8.0 (25.7)	8.4 (28.4)	39.8 (16.2)	25.7–48.1 (10.5–19.6)
Eastern Mediterranean	0.8 (3.3)	2.1 (7.8)	2.9 (10.5)	3.1 (12.1)	2.9 (13.1)	2.4 (13.7)	2.1 (14.0)	16.2 (10.5)	1.9–27.7 (1.2–17.8)
Europe	0.6 (3.4)	2.1 (8.2)	3.5 (11.4)	4.2 (13.4)	4.5 (14.7)	4.8 (15.5)	4.9 (16.1)	24.7 (11.2)	8.2–36.4 (3.7–16.5)
South-East Asia	0.1 (0.2)	0.3 (0.4)	0.3 (0.4)	0.3 (0.4)	0.3 (0.4)	0.2 (0.4)	0.2 (0.4)	1.8 (0.4)	0.1–8.3 (0.0–1.7)
Western Pacific	0.4 (1.0)	1.1 (1.9)	1.7 (2.2)	1.6 (2.3)	1.5 (2.3)	1.7 (2.4)	1.8 (2.4)	9.8 (2.0)	0.1–66.9 (0.0–13.5)
Total	3.0 (1.1)	9.0 (3.2)	13.6 (4.6)	15.8 (5.8)	16.6 (6.8)	17.1 (7.3)	17.4 (7.8)	92.5 (5.1)	54.6–154.6 (3.0–8.5)
**Male**									
Africa	< 0.1^b^ (0.1)	< 0.1^b^ (0.1)	< 0.1^b^ (0.1)	< 0.1^b^ (0.1)	< 0.1^b^ (0.1)	< 0.1^b^ (0.1)	< 0.1^b^ (0.1)	0.2 (0.1)	< 0.1^c^–3.7 (0.0–1.7)
Americas	0.9 (3.0)	3.1 (8.5)	4.9 (13.4)	6.2 (17.7)	7.0 (21.5)	7.5 (24.8)	7.9 (27.7)	37.4 (15.4)	23.8–45.4 (9.8–18.7)
Eastern Mediterranean	0.8 (3.3)	2.2 (7.8)	3.1 (10.5)	3.4 (12.1)	3.1 (13.1)	2.7 (13.7)	2.3 (14.0)	17.7 (10.5)	2.1–30.2 (1.2–18.0)
Europe	0.7 (3.5)	2.6 (9.7)	4.7 (14.9)	6.1 (19.4)	7.1 (23.1)	8.0 (26.3)	8.5 (29.0)	37.7 (16.9)	24.3–45.7 (10.9–20.5)
South-East Asia	0.2 (0.2)	0.3 (0.4)	0.3 (0.4)	0.3 (0.4)	0.3 (0.4)	0.3 (0.4)	0.2 (0.4)	1.9 (0.4)	0.2–8.8 (0.0–1.7)
Western Pacific	0.2 (0.5)	0.6 (0.9)	0.8 (1.0)	0.7 (1.0)	0.7 (1.0)	0.8 (1.0)	0.8 (1.0)	4.7 (0.9)	< 0.1^c^–59.7 (0.0–11.4)
Total	2.8 (0.9)	8.9 (3.0)	13.9 (4.5)	16.8 (6.0)	18.1 (7.2)	19.2 (8.0)	19.8 (8.7)	99.4 (5.3)	64.2–167.6 (3.4–8.9)

UI: uncertainty interval; WHO: World Health Organization.

^a^ 95% UI of the total no. of infected people in millions (95% UI of percentage prevalence).

^b^ Numbers are < 50 000 ≥ 10 000.

^c^ Numbers are < 10 000.

Notes: Numbers are the year 2016 estimated number of people living with genital herpes simplex virus type 1 infection. Prevalences are the percentage of infected people in the age- sex- and region-specific population. Numbers do not always sum exactly to the totals due to rounding. Regions are World Health Organization definitions.

When considering HSV type 1 infection at any site, we estimated 3752.0 million people (95% UI: 3555.5 million–3854.6 million) of the world’s population 0–49 years of age were infected, a prevalence of 66.6% (95% UI: 63.1–68.4; data repository).^23^ The regional pooled prevalence values and model fits are available in the data repository.^23^ Note that the number of people with oral and genital HSV type 1 infections do not sum exactly to the number with HSV type 1 infection at any site, as we assumed a small proportion of people can be infected at both sites simultaneously. The estimates were highly sensitive to our assumption that anyone aged 15 years of age and older who does not have an existing infection can acquire genital HSV type 1. If only those individuals who engaged in oral sex in the last year are at risk of acquiring genital HSV type 1 infection, then we estimate 122.3 million (3.3%) of those 15–49 years of age had prevalent genital HSV type 1 infections in 2016 (data repository).^23^

Taken together, an estimated 596.0 million–655.7 million people, 16.0–17.6% of the world’s population 15–49 years of age, had genital HSV type 1 or HSV type 2 or both, based on 122.3 million–192.0 million genital HSV type 1 infections. 

Applying the prevalence in those aged 45–49 years of age to population numbers for those aged 50–99 years of age, we estimated that globally, a total of 1290.1 million and 344.5 million people aged 50–99 years were infected with HSV type 1 (any site) and HSV type 2, respectively, bringing the totals to 4850.1 million and 836.0 million, respectively (data repository).^23^

The global prevalence of HSV type 2 for 2016 (13.2%; 95% UI: 11.5–16.3) was estimated to be somewhat higher than that estimated for 2012 (11.3%; 95% UI: 7.4–18.4),^2^ although the 95% UI overlapped. Applying equal population numbers by age, sex, WHO region and estimate year, the observed increase in global HSV type 2 prevalence between 2012 and 2016 remained but was somewhat diminished (13.7% versus 15.2%). This pattern was seen across all regions and especially for females, except for the Eastern Mediterranean Region, where a decrease was observed.

### Incidence of infection

We estimated that 23.9 million (95% UI: 21.0 million–29.5 million) people 15–49 years of age became infected with HSV type 2 in 2016, an incidence of 0.6% (95% UI: 0.6–0.8; Table 4). Of these, 14.7 million (95% UI: 12.4 million–18.1 million) were women and 9.2 million (95% UI: 7.4 million–13.6 million) were men. The number was highest in the WHO African Region, and there was an overall trend of decreasing incidence with age, as prevalence increased. However, the pattern was less marked for those settings where prevalence increased steadily with age (data repository).^23^

**Table 4:** Global and regional estimates of the incidence of herpes simplex virus type 2 infection by age and sex, 2016

WHO region by sex	No. of infected people in millions (population incidence, %) by age								95% UI^a^
	15–19 years	20–24 years	25–29 years	30–34 years	35–39 years	40–44 years	45–49 years	Total	
**Total**	5.2 (0.9)	4.4 (0.8)	3.9 (0.6)	3.2 (0.6)	2.7 (0.5)	2.4 (0.5)	2.1 (0.5)	23.9 (0.6)	21.0–29.5 (0.6–0.8)
**Female**									
Africa	1.8 (3.4)	1.1 (2.5)	0.7 (1.9)	0.5 (1.4)	0.3 (1.0)	0.2 (0.7)	0.1 (0.5)	4.6 (2.0)	4.1–4.9 (1.7–2.1)
Americas	0.5 (1.4)	0.5 (1.3)	0.4 (1.2)	0.4 (1.1)	0.3 (1.0)	0.3 (0.9)	0.3 (0.9)	2.7 (1.1)	2.2–3.3 (0.9–1.4)
Eastern Mediterranean	0.1 (0.4)	0.1 (0.4)	0.1 (0.4)	0.1 (0.4)	0.1 (0.4)	0.1 (0.4)	0.1 (0.4)	0.7 (0.4)	0.3–1.5 (0.2–0.9)
Europe	0.1 (0.5)	0.1 (0.5)	0.2 (0.5)	0.2 (0.5)	0.2 (0.5)	0.1 (0.5)	0.1 (0.5)	1.0 (0.5)	0.5–2.0 (0.2–1.0)
South-East Asia	0.5 (0.5)	0.4 (0.5)	0.4 (0.5)	0.4 (0.5)	0.3 (0.5)	0.3 (0.5)	0.2 (0.5)	2.5 (0.5)	1.1–5.2 (0.2–1.0)
Western Pacific	0.4 (0.7)	0.5 (0.7)	0.5 (0.7)	0.5 (0.7)	0.4 (0.6)	0.4 (0.6)	0.5 (0.6)	3.1 (0.7)	2.0–4.6 (0.4–1.0)
Total	3.4 (1.2)	2.7 (1.0)	2.4 (0.8)	1.9 (0.7)	1.6 (0.6)	1.4 (0.6)	1.3 (0.6)	14.7 (0.8)	12.4–18.1 (0.7–1.0)
**Male**									
Africa	0.9 (1.7)	0.7 (1.6)	0.5 (1.4)	0.4 (1.3)	0.3 (1.2)	0.2 (1.1)	0.2 (1.0)	3.3 (1.4)	2.5–4.1 (1.1–1.7)
Americas	0.2 (0.6)	0.2 (0.6)	0.2 (0.6)	0.2 (0.6)	0.2 (0.6)	0.2 (0.5)	0.2 (0.5)	1.4 (0.6)	0.9–2.1 (0.4–0.9)
Eastern Mediterranean	< 0.1^b^ (0.2)	< 0.1^b^ (0.2)	< 0.1^b^ (0.2)	< 0.1^b^ (0.2)	< 0.1^b^ (0.2)	< 0.1^b^ (0.2)	< 0.1^b^ (0.1)	0.3 (0.2)	0.1–1.3 (0.0–0.7)
Europe	0.1 (0.3)	0.1 (0.3)	0.1 (0.3)	0.1 (0.3)	0.1 (0.3)	0.1 (0.2)	0.1 (0.2)	0.5 (0.3)	0.2–1.1 (0.1–0.5)
South-East Asia	0.4 (0.4)	0.4 (0.4)	0.3 (0.4)	0.3 (0.4)	0.3 (0.4)	0.2 (0.4)	0.2 (0.4)	2.0 (0.4)	0.7–5.9 (0.1–1.1)
Western Pacific	0.2 (0.4)	0.2 (0.4)	0.3 (0.3)	0.2 (0.3)	0.2 (0.3)	0.2 (0.3)	0.3 (0.3)	1.7 (0.3)	0.8–3.6 (0.2–0.7)
Total	1.8 (0.6)	1.6 (0.5)	1.5 (0.5)	1.3 (0.5)	1.1 (0.4)	1.0 (0.4)	0.9 (0.4)	9.2 (0.5)	7.4–13.6 (0.4–0.7)

UI: uncertainty interval; WHO: World Health Organization.

^a^ 95% UI of the total no. of infected people in millions (95% UI of percentage incidence).

^b^ Numbers are < 50 000 ≥ 10 000.

Notes: Numbers are the estimated number of people newly infected with herpes simplex virus type 2 during 2016. Incidences are the percentage of infected people in the age- sex- and region-specific population. Numbers do not always sum exactly to the totals due to rounding. Regions are World Health Organization definitions.

An estimated 120.4 million (95% UI: 114.3 million–130.1 million) people 0–49 years of age acquired HSV type 1 infection at any site, an incidence of 2.1% (95% UI: 2.0–2.3; Table 5). The number was highest in the African Region, and decreased with age, most notably in regions where prevalence saturated at younger ages (data repository).^23^ The available empirical incidence data suggested that the force of infection may vary with age (data repository),^23^ but the data were too limited to draw further conclusions.

**Table 5:** Global and regional estimates of the incidence of herpes simplex virus type 1 infection (at any site) by age and sex, 2016

WHO region by sex	No. of infected people in millions (population incidence, %) by age											95% UI^a^
	0–4 years	5–9 years	10–14 years	15–19 years	20–24 years	25–29 years	30–34 years	35–39 years	40–44 years	45–49 years	Total	
**Total**	81.4 (12.3)	19.1 (3.0)	7.0 (1.2)	3.8 (0.7)	2.7 (0.5)	2.1 (0.3)	1.5 (0.3)	1.2 (0.2)	0.9 (0.2)	0.7 (0.2)	120.4 (2.1)	114.3–130.1 (2.0–2.3)
**Female**												
Africa	16.7 (21.4)	1.6 (2.4)	0.2 (0.3)	< 0.1^b^ (0.0)	< 0.1^c^ (0.0)	< 0.1^c^ (0.0)	< 0.1^c^ (0.0)	< 0.1^c^ (0.0)	< 0.1^c^ (0.0)	< 0.1^c^ (0.0)	18.5 (4.2)	16.1–22.0 (3.6–5.0)
Americas	1.0 (3.0)	0.9 (2.5)	0.8 (2.1)	0.7 (1.8)	0.6 (1.6)	0.5 (1.3)	0.4 (1.1)	0.3 (1.0)	0.3 (0.8)	0.2 (0.7)	5.6 (1.6)	5.4–5.8 (1.5–1.7)
Eastern Mediterranean	3.1 (7.8)	1.7 (4.7)	0.9 (2.8)	0.5 (1.7)	0.3 (1.0)	0.2 (0.6)	0.1 (0.4)	< 0.1^b^ (0.2)	< 0.1^b^ (0.1)	< 0.1^b^ (0.1)	6.8 (2.5)	5.3–8.0 (1.9–2.9)
Europe	1.7 (6.5)	1.1 (4.2)	0.7 (2.7)	0.4 (1.7)	0.3 (1.1)	0.2 (0.7)	0.2 (0.5)	0.1 (0.3)	0.1 (0.2)	< 0.1^b^ (0.1)	4.8 (1.7)	4.4–5.0 (1.5–1.8)
South-East Asia	10.5 (12.7)	2.2 (2.5)	0.4 (0.5)	0.1 (0.1)	< 0.1^b^ (0.0)	< 0.1^b^ (0.0)	< 0.1^c^ (0.0)	< 0.1^c^ (0.0)	< 0.1^c^ (0.0)	< 0.1^c^ (0.0)	13.2 (1.7)	12.6–14.2 (1.7–1.9)
Western Pacific	7.0 (12.3)	2.3 (4.2)	0.7 (1.4)	0.3 (0.5)	0.1 (0.2)	< 0.1^b^ (0.1)	< 0.1^b^ (0.0)	< 0.1^c^ (0.0)	< 0.1^c^ (0.0)	< 0.1^c^ (0.0)	10.5 (1.6)	9.8–13.1 (1.5–2.0)
Total	40.0 (12.6)	9.8 (3.2)	3.7 (1.3)	2.0 (0.7)	1.3 (0.5)	0.9 (0.3)	0.7 (0.2)	0.5 (0.2)	0.3 (0.1)	0.3 (0.1)	59.4 (2.2)	56.3–63.9 (2.0–2.3)
**Male**												
Africa	17.2 (21.4)	1.7 (2.4)	0.2 (0.3)	< 0.1^b^ (0.0)	< 0.1^c^ (0.0)	< 0.1^c^ (0.0)	< 0.1^c^ (0.0)	< 0.1^c^ (0.0)	< 0.1^c^ (0.0)	< 0.1^c^ (0.0)	19.0 (4.3)	16.5–22.6 (3.7–5.1)
Americas	0.9 (2.4)	0.8 (2.1)	0.7 (1.9)	0.6 (1.6)	0.6 (1.4)	0.5 (1.3)	0.4 (1.1)	0.3 (1.0)	0.3 (0.9)	0.2 (0.8)	5.2 (1.5)	4.9–5.5 (1.4–1.6)
Eastern Mediterranean	3.3 (7.8)	1.8 (4.7)	0.9 (2.8)	0.5 (1.7)	0.3 (1.0)	0.2 (0.6)	0.1 (0.4)	0.1 (0.2)	< 0.1^b^ (0.1)	< 0.1^b^ (0.1)	7.2 (2.4)	5.7–8.4 (1.9–2.9)
Europe	0.9 (3.1)	0.7 (2.6)	0.6 (2.2)	0.5 (1.9)	0.5 (1.6)	0.4 (1.4)	0.4 (1.1)	0.3 (1.0)	0.3 (0.8)	0.2 (0.7)	4.7 (1.6)	4.3–5.1 (1.5–1.7)
South-East Asia	11.4 (12.7)	2.4 (2.5)	0.5 (0.5)	0.1 (0.1)	< 0.1^b^ (0.0)	< 0.1^c^ (0.0)	< 0.1^c^ (0.0)	< 0.1^c^ (0.0)	< 0.1^c^ (0.0)	< 0.1^c^ (0.0)	14.4 (1.8)	13.8–15.5 (1.7–1.9)
Western Pacific	7.7 (12.0)	2.0 (3.2)	0.5 (0.9)	0.1 (0.2)	< 0.1^b^ (0.1)	< 0.1^b^ (0.0)	< 0.1^c^ (0.0)	< 0.1^c^ (0.0)	< 0.1^c^ (0.0)	< 0.1^c^ (0.0)	10.5 (1.5)	9.4–15.5 (1.3–2.2)
Total	41.3 (12.1)	9.3 (2.8)	3.4 (1.1)	1.9 (0.6)	1.4 (0.5)	1.1 (0.4)	0.9 (0.3)	0.7 (0.3)	0.5 (0.2)	0.4 (0.2)	61.0 (2.1)	57.6–66.8 (2.0–2.3)

UI: uncertainty interval; WHO: World Health Organization.

^a^ 95% UI of the total no. of infected people in millions (95% UI of percentage incidence).

^b^ Numbers are < 50 000 ≥ 10 000.

^c^ Numbers are < 10 000.

Notes: Numbers are the estimated number of people newly infected with herpes simplex virus type 1 during 2016. Incidences are the percentage of infected people in the age- sex- and region-specific population. Incidence values of < 0.05% are rounded to 0.0%. Numbers do not always sum exactly to the totals due to rounding. Regions are World Health Organization definitions.

## Discussion

Our estimates updated to 2016 found around 491 million people living with HSV type 2 infection, 3583.5 million with oral HSV type 1 infection and 122 million –192 million with genital HSV type 1 infection, in those up to 49 years of age. An estimated 596 million–656 million people were genitally infected with either HSV type 1 or 2, meaning that HSV has a substantial effect on the sexual and reproductive health of millions of people worldwide. HSV type 2 infection disproportionately affected women and the WHO African Region. It is concerning that around half of women aged 25–34 years of age in the African Region were infected with HSV type 2, as young women in this region are also at particularly high risk of acquiring HIV.^123^

These estimates for 2016 were informed by extensive literature reviews, with 474 and 223 prevalence data points for HSV type 2 and type 1, respectively, contributing to the estimates. For this update, we also made separate estimates for the numbers of people with oral HSV type 1 infection. Our estimates provide a global picture of the overall numbers of HSV infections and can be built upon to better understand the global burden of HSV-associated disease.

The estimates have some limitations, however. First, our pooled estimates rely on the accuracy of the data, which inform them and assume that the contributing studies are representative of their respective regions. Despite an increased number of studies contributing data compared with previous estimates, the number of contributing countries was lower in 2016. To help mitigate these issues, we used broad literature search terms and did not restrict the search by language. We adjusted the reported prevalence for assay sensitivity and specificity, since lack of adjustment tends to inflate HSV prevalence, and we generated estimate bounds to reflect the uncertainty in prevalence reported by publications. We also assumed a constant force of infection by age. However, we applied the force of infection only to those who were susceptible, allowing the number of infected people to decrease with age, and the fitting process also allowed the prevalence to saturate below 100% where suggested by the data. Nonetheless, future modelling analyses would be useful to explore how the limited available empirical incidence data could further inform estimates of infection. In the meantime, our estimates provide a snapshot of prevalence and the limitations of incidence estimates have less impact, as incidence only needs to be projected ahead by a single year.

Second, our estimates for genital HSV type 1 infection are particularly uncertain, as reflected in the wide uncertainty intervals. HSV type 1 prevalence data are lacking among children for all regions and across all ages for the WHO African and South-East Asia Regions. Accurate fitting to prevalence is important for predicting the potential for acquiring genital infection when a person commences sexual activity. Our model fits suggested that in some regions, few HSV type 1 infections are acquired in adulthood, resulting in low estimates of genital HSV type 1 infection. However, since there were limited data to inform the model fits, the numbers could be higher than estimated. Conversely, we applied a relatively high value to the proportion of incident HSV type 1 infections that are genital during adulthood. Although we pooled this value across contributing studies, the incidence of genital HSV type 1 was based on data from only four longitudinal studies, all from the USA and in sexually active populations that may not be representative of other regions. In addition, the value was calculated by assuming that oral and genital HSV type 1 infections are equally likely to be symptomatic. The proportion of infections that were genital may vary across regions due to variations in the practice of oral sex, the main route of transmission of HSV type 1 genital herpes, as well as variations in the background prevalence of HSV type 1 infection.^5^ Thus, there is also potential for overestimation of the contribution of genital infections to all HSV type 1 infections. Our sensitivity analysis showed how genital HSV type 1 infection estimates might change if fewer people were able to acquire infection.

Third, our infection estimates do not translate into direct estimates of symptoms or disease. Of the more than half a billion people estimated to be genitally infected with either HSV type 1 or type 2 for example, many infections will be asymptomatic (or at least, not recognized as genital herpes), particularly those due to genital HSV type 1. Even in the absence of symptoms, infected people can transmit HSV to sex partners or neonates and may have a higher risk of acquiring HIV, as documented for HSV type 2.^13^

Fourth, time trends between estimates from different years should be interpreted cautiously. The estimated global prevalence of HSV type 2 for 2016 was somewhat higher than for 2012, although not significantly so. In addition, we used population data for a single year to make our estimates, but there was a large overlap in the available data between estimate years. At the same time, there were changes in the countries and types of populations contributing data between 2012 and 2016. Furthermore, both overall prevalence and numbers of people infected are a function of the underlying demography of a region, and there has been a global shift towards an ageing population. This shift will increase the overall prevalence of infection even in the absence of a change in the force of infection, since HSV infection is lifelong, as shown by our analysis of age-standardized prevalence. Time trends can be investigated in future research through analyses of study-level data.

Lastly, by restricting the analysis to those younger than 50 years of age, we have underestimated the total burden of infection. Older age groups not only have highest prevalence of infection, but likely also contribute an important burden of disease in terms of continuing recurrences.^124^ We used this cut-off because individuals 15–49 years of age is the most important age group in terms of risk of sexual transmission and sexual and reproductive health outcomes, and because data on HSV prevalence in older people are limited. Using this age range also allows us to align our data with other sexually transmitted infection estimates produced by WHO, which are done for individuals 15–49 years of age^125^ To explore the potential for underestimation, we extended the age range and found that globally for 2016, 4850.1 million and 836.0 million people aged up to 99 years may have HSV type 1 and type 2 infection, respectively. Our HSV type 2 estimate is similar to the 956 million (95% UI: 847 million–1087 million) estimated by the 2017 GBD study, which included older ages.^17^ The GBD study uses a Bayesian model with HSV type 2 prevalence data identified by a basic search string supplemented by data from our more comprehensive searches.^2^^,^^16^ GBD HSV type 2 infection estimates were not adjusted for test underperformance, which tends to overestimate prevalence.^2^ Differences in regional groupings will also influence global totals.

Although not all infections lead to symptoms, our estimate of more than half a billion people with genital HSV infection translates into a large burden of disease worldwide. Current methods of prevention against HSV infection, such as the use of condoms, or antiviral drugs by the infecting partner, are inadequate.^19^ These estimates for 2016 can inform the development and subsequent targeting of interventions to maximize the impact on morbidity and mortality, especially in low- and middle-income countries.
